# Early tirofiban versus heparin for bridging dual antiplatelet therapy in patients undergoing coronary endarterectomy combined with coronary artery bypass grafting: a multicenter randomized controlled trial protocol (the THACE-CABG trial)

**DOI:** 10.1186/s13063-023-07737-8

**Published:** 2024-01-15

**Authors:** Liang Chen, Ming-Xin Gao, Xin Du, Chi Wang, Wen-Yuan Yu, Hong-Li Liu, Xiao-Hang Ding, Bo-Lin Wang, Kui Zhang, Dong Xu, Zhen Han, Bao-Dong Xie, Ran Dong, Yang Yu

**Affiliations:** 1 Department of Cardiovascular Surgery, Beijing Anzhen Hospital, Capital Medical University, No.2 Anzhen Road, Chaoyang District, Beijing, China; 2 Department of Cardiovascular Surgery, Shanghai Deltahealth Hospital, Shanghai, China; 3 Department of Cardiology, Beijing Anzhen Hospital, Capital Medical University, Beijing, China; 4 National Clinical Research Centre for Cardiovascular Diseases, Beijing, China; 5 The George Institute for Global Health, University of New South Wales, Sydney, NSW Australia; 6 Heart Health Research Center, Beijing, China; 7 Department of Cardiovascular Surgery, Beijing Tiantan Hospital, Capital Medical University, Beijing, China; 8 Department of Cardiovascular Surgery, Peking University Shenzhen Hospital, Shenzhen, Guangdong Province China; 9 Department of Cardiovascular Surgery, The First Affiliated Hospital of Harbin Medical University, Heilongjiang Province, Harbin, China

**Keywords:** Tirofiban, Heparin, Coronary endarterectomy, Coronary artery bypass grafting, Antithrombotic therapy, Major cardiovascular and cerebrovascular events, Randomized controlled trial

## Abstract

**Background:**

For complete revascularization, patients with diffuse coronary artery disease should have a coronary endarterectomy and a coronary artery bypass graft (CE-CABG). Sadly, CE can lead to a lack of endothelium, which raises the risk of thrombotic events. Even though daily dual antiplatelet therapies (DAPT) have been shown to reduce thrombotic events, the risk of perioperative thrombotic events is high during the high-risk period after CE-CABG, and there is no consistent protocol to bridge DAPT. This trial aims to compare safety and efficacy between tirofiban and heparin as DAPT bridging strategies after CE-CABG.

**Methods:**

In phase I, 266 patients undergoing CE-CABG will be randomly assigned to tirofiban and heparin treatment groups to compare the two treatments in terms of the primary safety endpoint, chest tube drainage in the first 24 h. If the phase I trial shows tirofiban non-inferiority, phase II will commence, in which an additional 464 patients will be randomly assigned. All 730 patients will be studied to compare major cardiovascular and cerebrovascular events (MACCEs) between the groups in the first 30 days after surgery.

**Discussion:**

Given the possible benefits of tirofiban administration after CE-CABG, this trial has the potential to advance the field of adult coronary heart surgery.

**Trial registration:**

chictr.org.cn, ChiCTR2200055697. Registered 6 January 2022. https://www.chictr.org.cn/com/25/showproj.aspx?proj=149451. Current version: 20,220,620.

## Administrative information

Note: the numbers in curly brackets in this protocol refer to SPIRIT checklist item numbers. The order of the items has been modified to group similar items (see http://www.equator-network.org/reporting-guidelines/spirit-2013-statement-defining-standard-protocol-items-for-clinical-trials/).

**Table:** 

Title {1}	Tirofiban versus heparin bridged with antiplatelet for the prevention of major cardiovascular and cerebrovascular events in patients undergoing coronary endarterectomy combined with coronary artery bypass grafting (THACE-CABG): study protocol for a multicenter randomized controlled trial
Trial registration {2a and 2b}.	www.chictr.org.cn: Trial identifier: ChiCTR2200055697 Registry name: Tirofiban versus heparin bridged with antiplatelet for the prevention of major cardiovascular and cerebrovascular events in patients undergoing coronary endarterectomy combined with coronary artery bypass grafting.
Protocol version {3}	Version 20,220,620
Funding {4}	The THACE-CABG trial is supported by grants from the Capital Health Research and Development of Special Fund (No. 2020–1-2061), Beijing Hospitals Authority’s Ascent Plan (Code: DFL20220605), Beijing Municipal Natural Science Foundation (No. 7214222), the Beijing Hospitals Authority Youth Program (grant number: QML20230603), and the Science and Technology Development Fund of Beijing Anzhen Hospital (NO. AZ2022).
Author details {5a}	Liang Chen^1,2†^, Ming-Xin Gao^1†^, Xin Du^3,4,5^, Chi Wang^5^, Wen-Yuan Yu^1^, Hong-Li Liu^1^, Xiao-Hang Ding^1^, Bo-Lin Wang^1^, Kui Zhang^1^, Dong Xu^6^, Zhen Han^7^, Bao-Dong Xie^8^, Ran Dong^1*^, Yang Yu^1*^ 1 Department of Cardiovascular surgery, Beijing Anzhen Hospital, Capital Medical University, Beijing, China 2 Department of Cardiovascular Surgery, Shanghai Deltahealth Hospital, Shanghai, China 3 Department of Cardiology, Beijing Anzhen Hospital, Capital Medical University 4 National Clinical Research Centre for Cardiovascular Diseases, Beijing, China 5 The George Institute for Global Health, University of New South Wales, Sydney, New South Wales, Australia 6 Heart Health Research Center, Beijing, China 7 Department of Cardiovascular Surgery, Beijing Tiantan Hospital, Capital Medical University, Beijing, China 8 Department of Cardiovascular surgery, Peking university Shenzhen hospital, Shenzhen, Guangdong Province, China 9 Department of Cardiovascular Surgery, The First Affiliated Hospital of Harbin Medical University, Harbin, Heilongjiang Province, China ^*^ Correspondence: Yang Yu, MD and Ran Dong, MD, No.2 Anzhen Road, Chaoyang District, Beijing, China. Email: heartyuyang@hotmail.com and dongran6618@hotmail.com † Equal contribution: These authors have contributed equally to this work..
Name and contact information for the trial sponsor {5b}	Yang Yu. Email: heartyuyang@hotmail.com
Role of sponsor {5c}	All authors made contributions to the development of the trial protocol and have been involved in drafting this manuscript or revising it critically or important intellectual content. LC and MXG designed the study and drafted the initial protocol; XD, CW and colleagues of HHRC gave much advice to revise it. MXG and YY applied for funding; LC, DX, ZH and BDX applied for ethical and regulatory approvals; LC, YWY, XHD, and BLW designed systems for collecting patient data; YY and RD are the project managers. All authors contributed in drafting this protocol, approved the final protocol and agreed to be held accountable for all aspects of this article. We are grateful to Shan Yan, Hou-Jian Zhao (Digital Health China Technologies Co., LTD) for technical support, who are not included in the author list. The funders in this study have no authority over any of the activities, including the study design; collection, management, analysis, and interpretation of data; writing of the report; and the decision to submit the report for publication.

## Introduction

### Background and rationale {6a}

Creation of distal surgical anastomoses can be difficult in patients with severe complex or diffuse coronary artery disease (CAD) undergoing coronary artery bypass grafting (CABG). Incomplete revascularization can affect patient survival and quality of life [1]. Coronary endarterectomy (CE) was first introduced in 1957 by Bailey et al. and is now being performed as an adjunct with CABG (CE-CABG) in patients with diffuse CAD to achieve complete revascularization [2]. Although long-term survival is comparable between CE-CABG and CABG alone, early surgical outcomes related to postoperative myocardial infarction (POMI) and mortality are worse in those undergoing CE-CABG [3–5]. These patients mainly experience graft occlusion owing to thrombosis in endarterectomized regions. During endarterectomy, the vascular intima is carefully dissected and removed, which exposes the subendothelial tissue to blood flow, causing fibrin platelet mural thrombus and activation of the coagulation cascade [6–8]. Dual antiplatelet therapy (DAPT) and postoperative anticoagulation may play a role in avoiding POMI after CE-CABG.

Current clinical guidelines recommend DAPT as soon as feasible after CABG and continuation for at least 12 months [9]. However, the use of antithrombotic medication within 24 h of CE-CABG is controversial. Reported rates of hemorrhage, POMI, and short-term mortality after CE-CABG vary substantially between studies [10–12]. Some centers have begun to use a heparin infusion 4 h after surgery in patients undergoing CE if the chest tube output is less than 50 to 100 mL/h. However, the incidence of POMI appears to be relatively high even with early heparin infusion [10, 11].

Tirofiban is a small-molecule peptide that selectively inhibits fibrinogen–platelet GP IIb/IIIa binding and reduces ischemic events in patients with acute coronary syndrome (ACS) undergoing percutaneous coronary intervention [13, 14]. Since perioperative use of GP IIb/IIIa inhibitors does not increase surgical bleeding after CABG [15–17], we hypothesized that early tirofiban administration before initiation of DAPT reduces the incidence of thrombosis and POMI after CE-CABG but does not increase bleeding incidence or severity.

### Objectives {7}

The primary goal of the THACE-CABG trial is to demonstrate the safety of early tirofiban administration after CE-CABG (phase I). If safety is demonstrated, its efficacy in reducing the incidence of major cardiovascular and cerebrovascular events (MACCEs) will be evaluated in comparison to early heparin administration (phase II).

### Trial design {8}

The THACE-CABG trial is a multicenter prospective randomized controlled trial. Patients will be randomized into one of two parallel groups at a 1:1 allocation ratio: an experimental group (tirofiban) and a control group (heparin). The safety portion of the study is a noninferiority trial with a primary endpoint of cumulative chest tube drainage in the first 24 h after surgery. The efficacy portion of the study is a superiority trial that will evaluate MACCEs in the 30 days after surgery as the primary endpoint. MACCEs include all-cause death, nonfatal MI, nonfatal acute ischemic stroke, and early revascularization.

## Methods: participants, interventions, and outcomes

### Study setting {9}

Beijing Anzhen Hospital, Capital Medical University, Beijing, China.

Beijing Tiantan Hospital, Capital Medical University, Beijing, China.

Peking University Shenzhen Hospital, Shenzhen, Guangdong Province, China.

The First Affiliated Hospital of Harbin Medical University, Harbin, Heilongjiang Province, China.

### Eligibility criteria {10}

Inclusion criteria

1. Age 18–80 years.
2. Primary diagnosis of non-ST elevation ACS or stable ischemic heart disease and appropriate candidate for isolated CABG.
3. Ability to understand the nature of the study and the study-related procedures and comply with them.
4. Undergoing CE-CABG.
5. Agreeable to provide written informed consent for surgery and study participation.

Exclusion criteria

1. Severe congestive heart failure (New York Heart Association class IV) or left ventricular ejection fraction ≤ 35%.
2. Tumor or suspected tumor.
3. Severe renal insufficiency (creatinine clearance < 30 mL/min) and/or chronic hemodialysis.
4. Cirrhosis or positive serum HBsAg/HBeAg, HCV-RNA, or anti HCV antibodies.
5. History of heparin/tirofiban allergy or thrombocytopenia after heparin/tirofiban.
6. Coagulation dysfunction, history of platelet abnormality or thrombocytopenia (preoperative platelet count < 150,000/mm^3^).
7. History of active internal hemorrhage, intracranial hemorrhage, or intracranial tumor, arteriovenous malformation, or aneurysm.
8. History of gastrointestinal or urogenital bleeding in the 6 months prior to randomization.
9. Stroke (any cause) or transient cerebral ischemia in the 6 months prior to randomization.
10. Pregnancy or breastfeeding.
11. Use of extra anticoagulation therapy, intra-aortic balloon pump, extracorporeal membrane oxygenation, or other circulatory support before, during, or in the first 2 h after surgery (individuals who experience excessive bleeding or require mechanical circulatory support and excessive anticoagulant medication within 2 h have intrinsic bleeding risks and are not candidates for further antithrombotic therapy).
12. CE-CABG surgery failed. CE-CABG failure. Graft flow and pulsatility index (PI) are measured at the end of the operation before protamine administration. If PI ≥ 5, the graft requires re-anastomosis; if still ≥ 5 after excluding the influence of operative technique, the operation is considered to have failed.
13. Chest tube bleeding > 150 mL in the first 2 h after surgery.
14. New-onset abnormal Q wave in the ECG within 2 h after surgery (indicates POMI; tirofiban or heparin are started 2 h after surgery for reducing POMI, thus individuals already having POMI within 2 h will be eliminated).

### Who will take informed consent? {26a}

A member of the study team will check eligibility and obtain written informed consent after explaining the study and answering any questions during a visit to the research site for the baseline assessment. No study operations will take place until consent is provided.

### Additional consent provisions for collection and use of participant data and biological specimens {26b}

Individuals who choose to participate in the study will be asked to give permission for long-term monitoring of their electronic medical records (with no additional patient contact). Permission will be obtained to utilize study data in other ways, such as for pooled analyses of anonymized data, natural history studies, meta-analyses, and health outcomes research.

## Interventions

### Explanation for the choice of comparators {6b}

After CE, the lack of endothelium leads to activation of the coagulation cascade [18–20]. Therefore, antithrombotic treatment is required [20]; however, no standard protocol exists. Heparin infusion followed by warfarin for several months has been recommended by several authors [21, 22]. Pre- or intraoperative clopidogrel dosing followed by postoperative aspirin and clopidogrel administration is another anticoagulation scheme which has been used [22]. In our hospital, we have used a postoperative heparin infusion followed by DAPT (aspirin and clopidogrel or ticagrelor) for many years. Tirofiban infusion followed by DAPT is another potential regimen which is purely antiplatelet in nature.

## Intervention description {11a}

### Standard procedure before randomization

Before surgery, patients hospitalized for isolated CABG will receive the following standard antithrombotic regimen: aspirin will be administered up to the day of the procedure; clopidogrel, ticagrelor, and prasugrel will be stopped at least 5, 5, and 7 days before, respectively.

CE-CABG will be performed by experienced surgeons via a median sternotomy utilizing either an on- or off-pump grafting technique. CE will be indicated for patients with: (1) a long, diffusely diseased coronary artery segment or severely calcified coronary artery, or (2) severe long-segment in-stent restenosis with or without major side branch occlusion [23]. CE is occasionally unanticipated prior to CABG surgery. CE-CABG is typically the last option for obtaining full revascularization. Patients who do not undergo CE have a better prognosis than those that do when antithrombotic medication is used according to current recommendations; therefore, they are not required to receive tirofiban therapy and will not be included.

Graft flow and PI will be assessed at the end of the operation before protamine administration using the VeriQ Flowmeter System (MediStim ASA, Horten, Norway), which is based on an ultrasonic transit time flow meter. If the PI is more than 5, the graft must be anastomosed again. If it remains above 5, the surgery is deemed a failure and the patient will be removed from the study.

After the procedure, protamine will be administered to reverse the heparin (100 IU heparin: 1 mg protamine) and the active coagulation time will be corrected to the pre-heparinization value of 5%. Chest tube blood loss will be evaluated 2 h after surgery; if < 150 mL, the patient will be randomized.

### Randomization and intervention

**Fig. 1:**
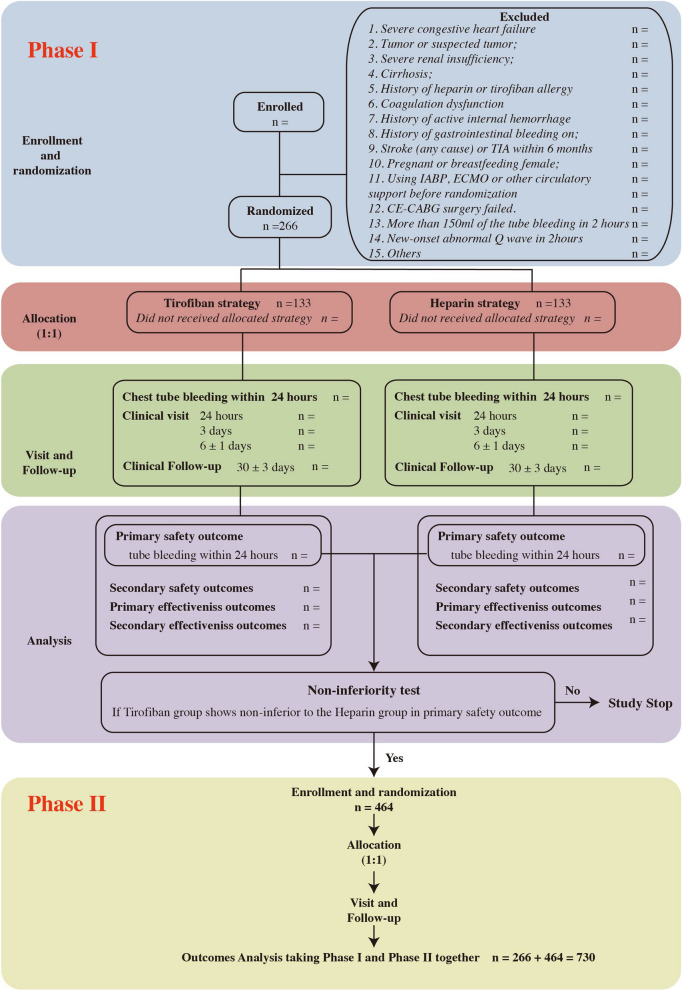
Study design. TIA, transient cerebral ischemia; IABP, intra-aortic balloon pump; ECMO, extracorporeal membrane oxygenation

Eligible patients will be randomized to either the heparin or tirofiban group in a 1:1 ratio utilizing an interactive online response system. Using the biased-coin minimization approach, a static randomization list will be created and applied taking three strata into consideration: research site, patient age group (< 70 and ≥ 70 years), and gender. Figure 1 depicts the enrollment and randomization procedures.

**Fig. 2:**
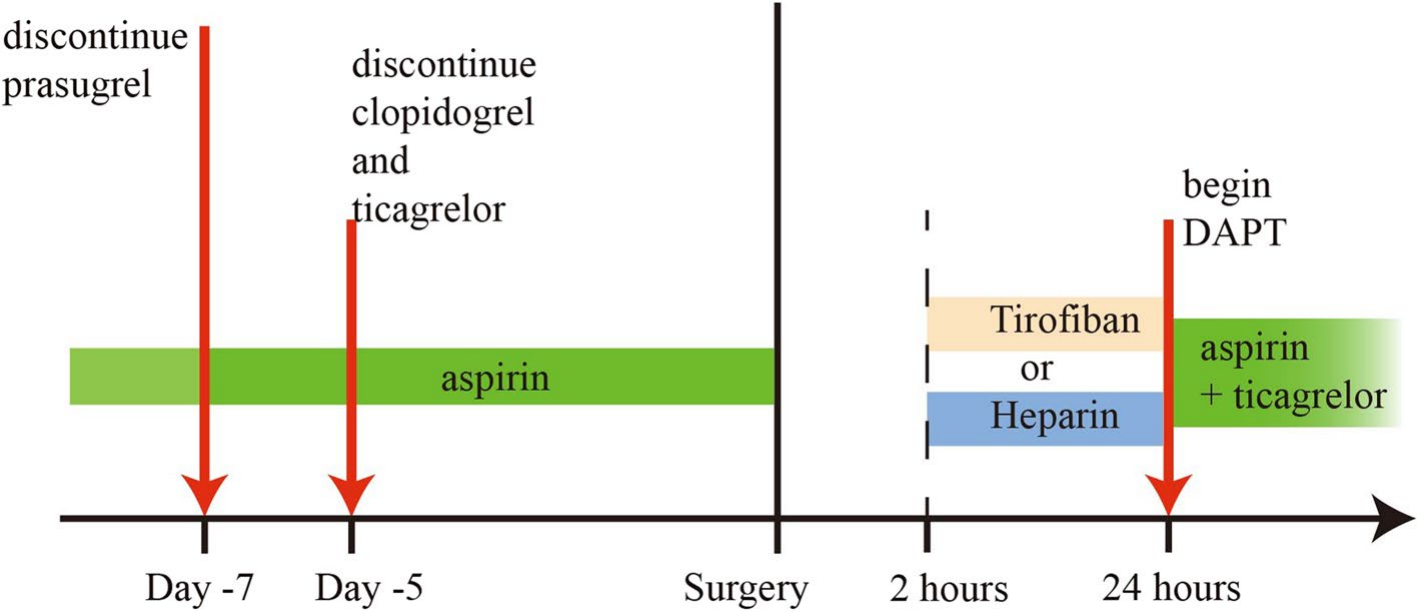
Timeline of antithrombotic drugs. DAPT, dual antiplatelet therapies

Two hours after randomization, heparin (100 IU/kg) will be administered intravenously in 5 mL of 0.9% sodium chloride every 4 h in the heparin group. In the tirofiban group, tirofiban (0.05 g/kg/min) will be infused over 22 h using a micropump starting within 30 min of randomization. To maintain treatment blinding, a 5-mL volume of 0.9% sodium chloride will be administered to patients in the tirofiban group, and patients in the heparin group will receive an infusion of 0.9% sodium chloride via micropump. Only the nurses who prepared the fluids will know which are genuine medications. Once heparin or tirofiban stop at 24 h, DAPT will be initiated the next day of surgery and continued daily (ticagrelor 90 mg twice daily plus aspirin 100 mg daily in patients with platelet count ≥ 9 × 10^9^ cells/L; aspirin alone daily in patients with platelet count < 9 × 10^9^ cells/L). For patients who are unable to be extubated within 24 h, heparin or tirofiban will be continued until the patient is extubated, at which point DAPT will start. The timeline of antithrombotic drugs is showed in Fig. 2.

To ensure patient safety, heparin and tirofiban doses will be adjusted according to routine blood and coagulation parameters obtained 4 and 10 h after treatment initiation. If the platelet count is < 90,000/mm^3^ after a repeat test, tirofiban infusion will be discontinued. If the activated partial thromboplastin time increases to 2.5 to 3 times the normal upper limit value, the heparin or tirofiban dose will be halved; if more than 3 times, they will be discontinued. If the fibrinogen level decreases to < 1.0 g/L, heparin and tirofiban infusions will be discontinued and fresh frozen plasma will be administered.

### Criteria for discontinuing or modifying allocated interventions {11b}

Participants may leave the experiment at any time for any reason as required by the Declaration of Helsinki. Although there are no particular withdrawal requirements, researchers can withdraw patients as necessary and note the cause. All participants will be followed for clinical outcomes unless permission is expressly revoked.

Pregnant women will be excluded. Menstrual history and human chorionic gonadotropin level will be examined in non-menopausal women. Pregnancy after enrollment will not be regarded as an adverse event or significant adverse event (SAE) in and of itself and will not be considered grounds for study exclusion; however, the patient will be given the opportunity to withdraw if she wishes.

During each study visit, patient safety will be addressed. Participants will receive information regarding who to contact in case of a SAE. Those who experience a SAE will be counseled to withdraw.

### Strategies to improve adherence to interventions {11c}

Three strategies to improve subject adherence to interventions will be implemented. First, a portion of the individuals’ health care and travel expenditures will be reimbursed. Second, physicians will offer free consultations. Finally, the researchers will communicate with the subjects at least once a week.

### Relevant concomitant care permitted or prohibited during the trial {11d}

Standard secondary CAD prevention measures will be implemented. Oral beta-blockers will be used before and after surgery to maintain heart rate between 60 and 80 beats per minute. To maintain low density lipoprotein concentration below 1.88 mol/L, an oral statin or combination of lipid-lowering medications will be prescribed. All episodes of care will be recorded in an electronic case report form (eCRF).

### Provisions for post-trial care {30}

Patients will be contacted every week in 30 days and once a year after trial to monitor for adverse effects, provide encouragement and motivation, answer questions, and assist with any problems.

### Outcomes {12}

#### Primary outcomes

The primary safety outcome (phase I) is total chest tube drainage output over 24 h. The primary efficacy outcome (phase II) is incidence of MACCEs in the first 30 days after surgery.

### Secondary outcomes

The secondary outcomes of the phase I trial are as follows: (1) the incidence of universal definition of perioperative bleeding (UDPB) in adult cardiac surgery class 3 or higher in the first 24 h after surgery [24] and (2) the incidence of Thrombolysis in Myocardial Infarction (TIMI) major bleeding in the first 30 days [25]. Table 1 shows the various classes of the universal definition of perioperative bleeding in adult cardiac surgery. TIMI major bleeding is defined as intracranial hemorrhage, > 5 g/dL decrease in hemoglobin concentration, or ≤ 15% absolute decrease in hematocrit.

**Table 1:** Bleeding categories according to the UDPB in adult cardiac surgery (if different categories indicate mixed definitions of bleeding, the worst definition applies)

Bleeding definition	Sternal closure delayed	Postoperative chest tube blood loss within 12 h (mL)	PRBC (unit)	FFP (unit)	PLT (unit)	Cryoprecipitate	PCCs	rFVIIa	Reexploration/tamponade
Class 0 (insignificant)	No	< 600	0^a^	0	0	No	No	No	No
Class 1 (mild)	No	601–800	1	0	0	No	No	No	No
Class 2 (moderate)	No	801–1000	2–4	2–4	Yes	Yes	Yes	No	No
Class 3 (severe)	Yes	1001–2000	5–10	5–10	N/A	N/A	N/A	No	Yes
Class 4 (massive)	N/A	> 2000	> 10	> 10	N/A	N/A	N/A	Yes	N/A

*UDPB *Universal definition for perioperative bleeding, *PRBC *Packed red blood cells, *FFP *Fresh frozen plasma, *PLT *Platelet concentrates, *PCCs *Prothrombin complex concentrates, *rFVIIa *Recombinant activated factor VII, *N/A *Not applicable

^a^Correction of preoperative anemia or hemodilution only; the number of PRBCs used should only be considered in the UDPB when accompanied by other signs of perioperative bleeding

The secondary outcomes of the phase II trial are as follows: (1) the incidence rates of the four different events comprising MACCEs and (2) the incidence of postoperative acute kidney injury. Both CABG and tirofiban have been associated with high risk of such injury.

### Definitions

All-cause death is defined as death from any cause. POMI is defined as MI within 48 h of CABG. MI is defined according to the fourth universal definition of MI: cardiac troponin concentration > 10 times the 99th percentile upper reference limit or > 20% increase in cardiac troponin concentration plus new pathologic Q waves on electrocardiography, graft occlusion on angiography, or new abnormal segmental ventricular wall motion on echocardiography. MI more than 48 h after surgery is defined as cardiac troponin concentration above the 99th percentile upper reference limit and at least one of the following: (1) new ischemia or new pathologic Q waves on electrocardiography, (2) imaging of new loss of viable myocardium or new regional wall motion abnormality in a pattern consistent with an ischemic cause, and (3) identification of a coronary thrombus on angiography [26].

All patients with stroke symptoms will be examined to rule out conditions that mimic stroke (e.g., seizure, conversion or somatoform disorder, migraine headache, and hypoglycemia) and then undergo urgent computed tomography and/or magnetic resonance imaging to confirm the diagnosis. Acute ischemic stroke is defined as a new neurologic deficit lasting more than 24 h in conjunction with imaging findings of cerebral infarction. Early revascularization is defined as any percutaneous or surgical revascularization performed for graft failure or ACS resulting from a lesion inside or adjacent to a graft within 30 days. Acute postoperative kidney injury is defined according to the Society of Thoracic Surgeons as the following: (1) serum creatinine ≥ 2 0.0 mg/dL or increase to twice the preoperative baseline concentration or (2) new requirement for dialysis [27].

### Participant timeline {13}

The participant timeline is shown in Table 2.

**Table 2:**
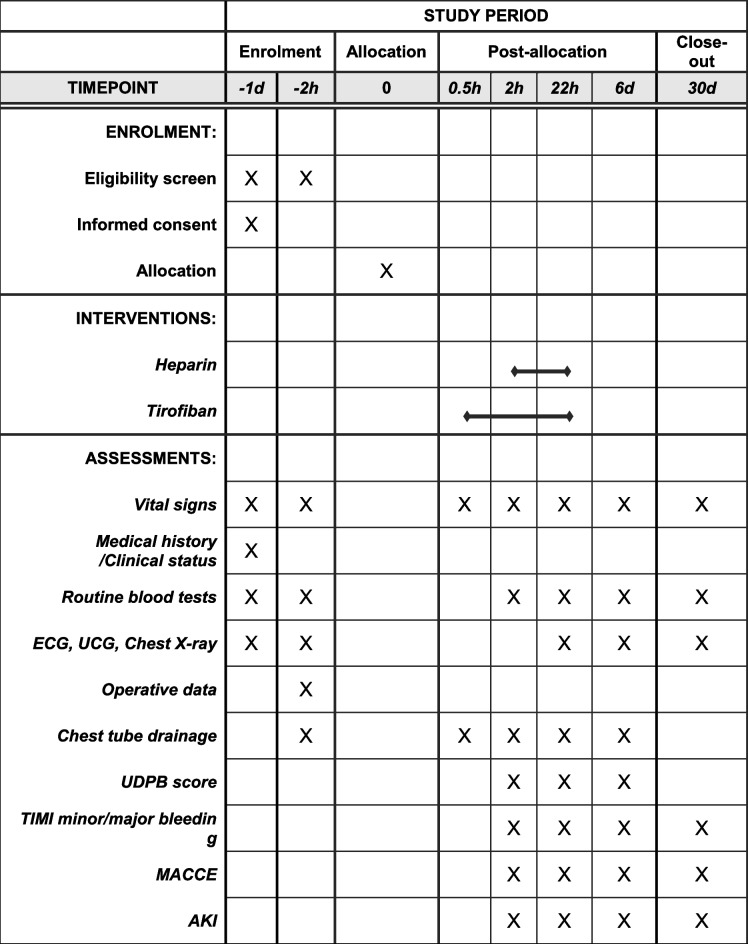
Schedule of enrolment, interventions and assessments

*ECG* Electrocardiogram, *UCG* echocardiography, *UDPB* Universal Definition for Perioperative Bleeding, *TIMI* Thrombolysis in Myocardial Infarction, *MACCE* Major adverse cardiovascular and cerebrovascular events, *AK* *I* Acute kidney injury

### Sample size {14}

We computed a sample size of 266 patients (133 per group) for the phase I trial, assuming a one-sided significance level of 2.5%, test power of 90%, chest tube drainage volume standard deviation of 500 mL, and non-inferiority limit of 200 mL. Thus, the non-inferiority will be demonstrated if the upper boundary of the 97.5% confidence interval for the mean difference is lower than 200 mL. A non-inferiority margin of 200 mL was arrived at by consensus among the investigators based on their clinical judgment and the data available at the time of trial design.

Based on data from the Anzhen Hospital database from 2020 to 2021, we estimate a 30-day MACCE incidence of approximately 40% in the control group (heparin). Using an absolute risk reduction of 25% as the anticipated intervention effect of tirofiban, acceptable alpha of 5%, and acceptable beta of 20%, 712 participants are required in each group. To allow for loss to follow-up, 730 participants will be recruited; therefore, after recruitment of the phase I trial participants, an additional 464 participants (232 per group) are required to satisfy the requirements for phase II.

### Recruitment {15}

The average number of annual CE-CABG procedures over the past three years at Anzhen hospital was approximately 600. Combined with the other two centers, achieving the required number of participants is feasible.

## Assignment of interventions: allocation

### Sequence generation {16a}

All eligible subjects will be allocated by clinical staff to either heparin group or tirofiban group in a 1:1 ratio at 2 h after CE-CABG using an interactive web response system (IWRS). A static randomization list is generated and implemented within the system which accounted for three strata: study site, patient age group (< 70, ≥ 70 years) and gender group (male, female) using the biased-coin minimization method. Each center will have a designated user of the interactive system. This user will generate a random sequence for patients who meet criteria for enrollment and communicate the allocation to the research nurse.

### Concealment mechanism {16b}

The random sequence will be immediately sent to a designated nurse who prepare the fluids and medications. For the tirofiban group, a 50-mL volume of tirofiban fluid and a 5-mL volume of 0.9% sodium chloride (labeled as heparin) will be prepared. For the heparin group, a heparin fluid and a 50-mL 0.9% sodium chloride (labeled as tirofiban) will be prepared.

### Implementation {16c}

Doctors and nurses working near the bed will have no idea which fluid is genuine medication. These fluids will be infused according their label names. Thus, all subjects, site staff, sponsor staff, assessors, and data analysts will be blinded to randomization outcomes.

## Assignment of interventions: blinding

### Who will be blinded {17a}

All subjects, site staff, sponsor staff, staff (with exceptions as indicated below), assessors, and data analysts will be blinded to randomization outcomes until the database is unlocked. Exceptions will include (1) nurses who prepare the fluids and medications (heparin, tirofiban, or just 0.9% sodium chloride), (2) computer programmers involved in randomization and drug management processes, and (3) the biostatistician creating reports for the Data and Safety Monitoring Board (DSMB).

### Procedure for unblinding if needed {17b}

The DSMB will periodically examine all safety and outcomes data. Appropriate information regarding adverse occurrences will be systematically gathered and presented to regulatory authorities.

Emergency unblinding will be performed only when necessary for subject safety. Only those individuals who are required to know treatment allocation will be given this information in such an event. If medically appropriate, all subjects will resume the study treatment after recovery and continue to do so until the end of the study.

## Data collection and management

### Plans for assessment and collection of outcomes {18a}

Data will be collected using a study-specific eCRF (Digital Health China Technologies Co., LTD, Beijing, China) and entered onto a paper case report form. Throughout the trial, data management personnel will validate the data. Data fields will be checked and any missing data or inconsistencies will be corrected before downloading into the study database. All identifying information will be hidden to protect patient confidentiality. All study personnel will have 24-h access to the study coordinating center. A data monitoring committee will monitor data accuracy and completeness, evaluate adverse events, and make the final decision to terminate the trial. When all data management and statistical data validation procedures have been completed, the database will be unlocked to perform the final analysis.

### Plans to promote participant retention and complete follow-up {18b}

There are no further plans for intervention engagement tactics beyond those that have already been described.

### Data management {19}

The DSMB will consist of a chair, co-chair, and members with recognized expertise in clinical trials, cardiovascular disease, and biostatistics who are not involved in the routine conduct of the THACE-CABG trial. The DSMB will be responsible for two main tasks. First, it will regularly assess the safety and effectiveness of the drugs under investigation by reviewing periodic updates provided during the course of the study. Second, it will provide recommendations to the trial’s chair and vice chair regarding the appropriate course of action, which may involve continuing, modifying, or terminating the study.

The DSMB’s primary responsibility is study oversight. Subject risks and benefits will be determined after an unblinded evaluation of aggregate data. The DSMB charter and meeting schedule will be finalized during the first meeting. A biostatistician, who will not be blinded for report preparation, will attend meetings to provide support. Conference attendees will be able to hear updates and ask questions of the trial’s chair and/or primary investigators during the open section of the conference.

### Confidentiality {27}

Only the lead investigator and necessary staff will have access to collected data. To protect patient confidentiality, each subject’s laboratory samples, completed forms, reports, and other data will be identified by a special participant ID number.

### Plans for collection, laboratory evaluation, and storage of biological specimens for genetic or molecular analysis in this trial/future use {33}

Not applicable: no biological specimens will be collected.

## Statistical methods

### Statistical methods for primary and secondary outcomes {20a}

Patient characteristics will be compared using the two-sample *t*-test or Wilcoxon rank sum test for continuous variables and the chi-square test or Fisher’s exact test for categorical variables. Variables that significantly differ between groups will be used as covariates for efficacy analysis.

Both intention-to-treat and per-protocol analyses will be conducted. The primary safety outcome will be compared between groups using a one-sided non-inferiority test. The primary efficacy outcome will be evaluated using the chi-square test or Fisher’s exact test.

### Interim analyses {21b}

Interim assessments and safety and efficacy monitoring will be conducted by the DSMB, which will review unblinded event rates. An outside statistician will perform interim data analyses independently for the DSMB.

A formal interim analysis is planned after the expected number of accumulated primary safety outcome events accrue (phase I). If the interim analyses show clear and consistent safety in both treatment arms, the DSMB may recommend commencement of the phase II trial. If the conditional probability of rejecting the non-inferiority hypothesis appears to be unacceptably high, the DSMB may consider postponing or ceasing the phase II trial.

### Methods for additional analyses (e.g., subgroup analyses) {20b}

A subgroup analysis of primary outcomes will be performed to compare CE in the left anterior descending coronary artery with CE in other coronary arteries. Subgroup analyses according to age, gender, diabetes, left ventricular ejection fraction, type of CE (open or closed), and graft type (vein or arterial) will also be performed.

Patient-reported outcomes and an analysis of cost-effectiveness will be reported at the end of the study. Projected changes in economic costs and health outcomes from broad use of tirofiban will be quantified. Market prices or shadow prices will be used to value costs. By counting and assessing the resources used, the costs of delivering the intervention will be determined.

### Methods in analysis to handle protocol non-adherence and any statistical methods to handle missing data {20c}

The analyses will not include participants who were randomly assigned but later determined to be ineligible. Additional types of non-compliance that can be identified from the research data, such as visits outside of predetermined times, will be evaluated and classified as significant or minor. Similarly, protocol violations will be rated as significant or minor. After participants with significant procedure violations are eliminated, sensitivity analyses will be conducted. Missing data will not be imputed for these analyses.

### Plans to give access to the full protocol, participant-level data, and statistical code {31c}

The corresponding author will provide trial datasets and algorithms upon reasonable request.

## Oversight and monitoring

### Composition of the coordinating center and trial steering committee {5d}

The trial steering committee comprises:

- Prof. Yang Yu (Chair)
- Prof. Ran Dong (Vice-chair)
- Prof. Dong Xu (Chief Investigator)
- Prof. Zhen Han (Chief Investigator)
- Prof. Bao-Dong Xie (Chief Investigator)
- Prof. Rong Han (Study Statistician)
- Prof. Xin Du (Study Project Manager)

### Composition of the data monitoring committee, its role and reporting structure {21a}

The Heart Health Research Center will serve as the data monitoring committee and receive all trial data. Data will be securely transmitted to the center for data entry and verification in accordance with their normal operating procedures.

### Adverse event reporting and harms {22}

Complete information regarding all SAEs (nature of the event, start and end dates, severity, link to the trial and/or trial protocols, and outcome) shall be noted in the medical record and eCRF within 24 h of occurrence. Such incidents will be followed up until satisfactorily resolved and stabilized. With reference to the study protocol and Reference Safety Information, the site principal investigator will utilize medical judgment to assess seriousness, causation, severity, and expectedness. The site team and main investigator will determine the relatedness and expectedness. According to the study protocol, the sponsor will validate data collection and SAEs. The sponsor will report safety information to the chief investigator or a delegate for ongoing risk/benefit assessment and collaborate with the chief investigator to submit an annual safety report to the research ethics committee until the end of the study, when an end of study form will be submitted.

The trial steering committee will assess safety in line with a predefined charter, reviewing recruiting and the overall status of the trial on a regular basis; it will also interact with the sponsor regarding safety problems. A relevant unexpected SAE will be reported to the sponsor, who will then notify the ethics committee. The study data center will offer an eCRF for central data collection of adverse events and SAEs which will be submitted to the trial steering committee every 6 months until the study is completed.

### Frequency and plans for auditing trial conduct {23}

An audit of all entries into the eCRF and number of participants will be conducted annually.

### Plans for communicating important protocol amendments to relevant parties (e.g., trial participants, ethical committees) {25}

Any protocol amendments will be authorized by the local ethics committee before being implemented and updated in the ChiCTR.org registration. All investigators and study participants will be informed.

Deviations from the protocol will be thoroughly documented using a report form.

### Dissemination plans {31a}

The findings of the study will be communicated largely through patient timeline and public forums, conference presentations, and scientific articles. The final manuscript will be reviewed and approved by all writers.

## Discussion

The THACE-CABG trial is a multicenter randomized controlled trial designed to assess the safety and efficacy of tirofiban infusion followed by DAPT in patients undergoing CE-CABG.

In non-inferiority trials, the null and alternative hypotheses are reversed compared to superiority trials; hence, the null hypothesis supposes a difference between the compared treatments. In other words, the alternative hypothesis supposes they do not differ or that one is non-inferior to the other.

In phase I of the THACE-CABG trial, the null (H0) and alternative (H1) hypotheses are as follows:

- H0: N—C > Δ (that is, chest tube drainage in the first 24 h after CE in the tirofiban group (N) is not non-inferior to that in the heparin group (C) by a pre-defined noninferiority margin of -Δ% or less, where Δ = 200 mL, or non-inferiority is not shown).
- H1: N—C ≤ Δ (that is, chest tube drainage in the first 24 h after CE in the tirofiban group is non-inferior to that in the heparin group).

**Fig. 3:**
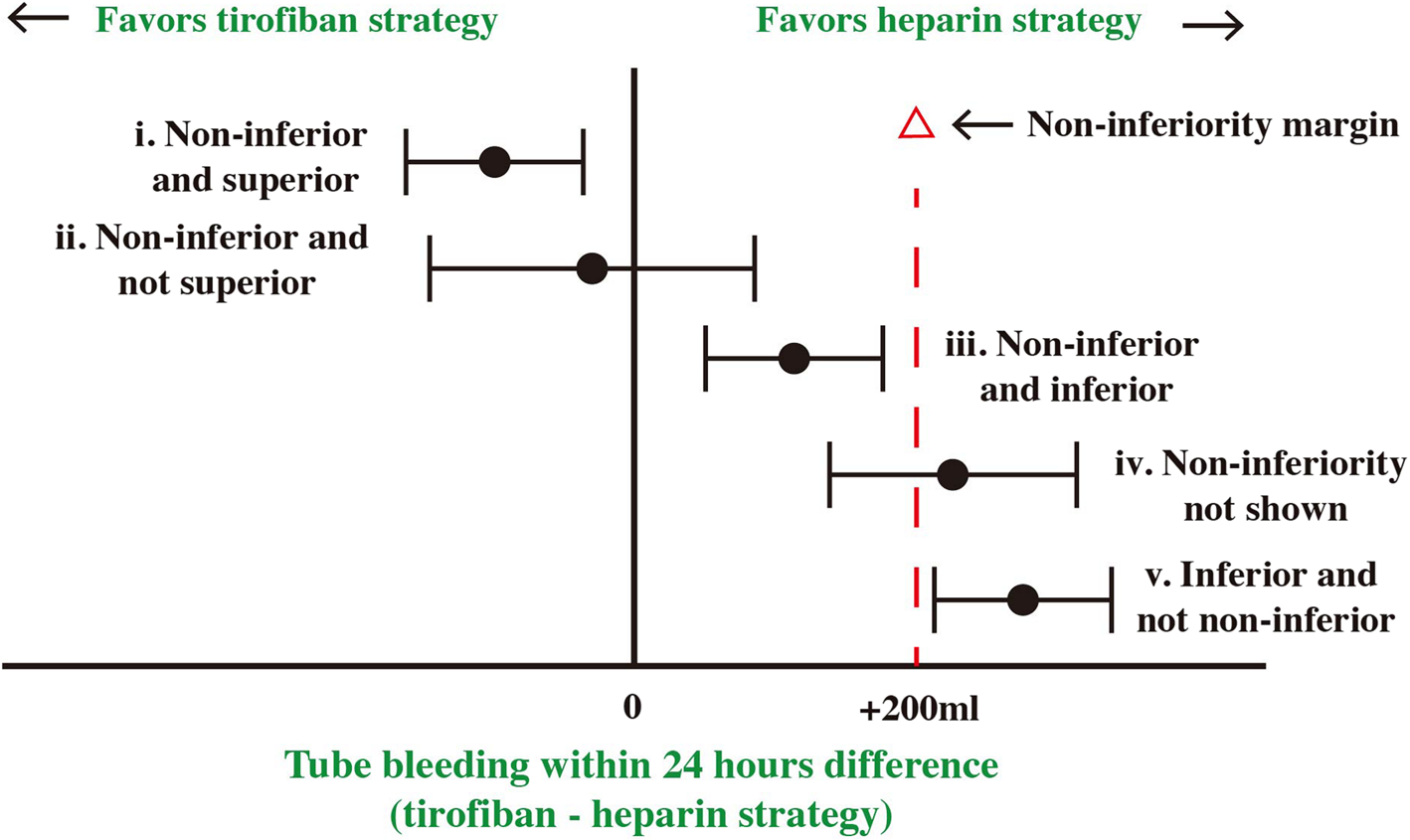
Possible outcomes for primary safety outcomes

In a non-inferiority study, five trial outcomes are possible (Fig. 3). For non-inferiority to be demonstrated (cases i, ii, and iii), the point estimate and 95% confidence interval of the difference in chest tube bleeding between the tirofiban and heparin groups have to be less than the non-inferiority margin of 200 mL. If non-inferiority is shown, three situations are possible: (1) non-inferiority and superiority, in which the point estimate and the confidence interval are less than 200 mL (case i); (2) non-inferiority and non-superiority, in which the point estimate lies below 200 mL and the confidence interval includes zero but not 200 mL (case ii); and (3) non-inferiority and inferiority, in which the point estimate and the entire confidence interval are between zero and 200 mL (case iii). Non-inferiority will not be shown if the confidence interval includes both the 200 mL non-inferiority margin and the zero line (case iv). Finally, inferiority will be established if the point estimate and entire confidence interval are above the 200 mL line (case v).

For the primary efficacy outcome, the hypothesis to be tested is whether incidence of MACCEs after CE-CABG is superior with tirofiban than with heparin. The study will be considered positive if statistical significance at the level of 0.05 (two-tailed) is achieved.

Risk of surgery is high in patients with ejection fraction < 35%. Bleeding events in these patients may be life-threatening. Therefore, such patients will not be included until tirofiban safety has been confirmed in others. Although this approach may introduce bias, it is warranted from a patient safety perspective.

In previous research, PI was proposed as a significant predictor of graft quality, showing the distal resistance of the graft vascular, with a suggested cutoff value of 5 [28]. A high PI suggests serious anastomotic stenosis, competitive flow, or severe distal coronary stenosis. All of these factors can contribute to thrombosis and graft failure. This thrombosis mechanism, however, differs from that of endothelial detachment, which is generated by blood stasis. To avoid misunderstanding, CE-CABG failure will be excluded. Our cohort is relatively selected, which could be a source of bias.

In conclusion, this is the first randomized controlled trial designed to compare safety and MACCEs between tirofiban and heparin administration after CE-CABG. In addition, we plan to use the trial’s database to conduct follow-up studies that evaluate long-term CE-CABG outcomes.

### Trial status

The study began recruitment on September 1, 2022, and is expected to end in late 2026. Current protocol: version 20,220,620.

## Data Availability

Trial data will be available upon reasonable request. All researchers will not have direct access to export patient data from the system. Researchers will be able to obtain data only with the agreement of the head investigator. Others involved will only be able to view data from other centers with the agreement of the project managers.

## Abbreviations

CE: Coronary endarterectomy
CABG: Coronary artery bypass grafting
CE-CABG: Coronary endarterectomy combined with coronary artery bypass grafting
DAPT: Dual antiplatelet therapies
MACCE: Major cardiovascular and cerebrovascular events
MI: Myocardial infarction
AIS: Acute ischemic stroke
CAD: Coronary artery disease
POMI: Postoperative myocardial infarction
PCI: Percutaneous coronary intervention
PI: Pulsatility index
ACT: Active coagulation time
TTFM: Ultrasonic transit time flow meter
UDPB: Universal definition for perioperative bleeding
TIMI: Thrombolysis in Myocardial Infarction
AE: Adverse event
SAE: Significant adverse event
eCRF: Electronic case report form
AKI: Acute kidney injury
UCG: Echocardiography
IWRS: Interactive web response system
DMC: Data Monitoring Committee
HHRC: The Heart Health Research Centre
RUSAE: Relevant Unexpected Serious Adverse Event
NSTE-ACS: Non-ST elevation acute coronary syndrome
SIHD: Stable ischemic heart disease
NYHA: New York Heart Association
HbsAg: Hepatitis B surface antigen
HBeAg: Hepatitis B e antigen
HCV: Hepatitis virus C
TIA: Transient cerebral ischemia
IABP: Intra-aortic balloon pump
ECMO: Extracorporeal membrane oxygenation
ECG: Electrocardiogram
PLT: Platelet concentrates
PCCs: Prothrombin complex concentrates
rFVIIa: Recombinant activated factor VII

## References

[1] Yi G, Youn Y-N, Joo H-C, Hong S, Yoo K-J (2013). Association of incomplete revascularization with long-term survival after off-pump coronary artery bypass grafting. J Surg Res.

[2] Bailey CP, May A, Lemmon WM (1957). Survival after coronary endarterectomy in man. J Am Med Assoc.

[3] Soylu E, Harling L, Ashrafian H, Casula R, Kokotsakis J, Athanasiou T (2014). Adjunct coronary endarterectomy increases myocardial infarction and early mortality after coronary artery bypass grafting: a meta-analysis. Interact Cardiovasc Thorac Surg.

[4] Song Y, Xu F, Du J, Zhang J, Feng W (2017). Coronary endarterectomy with coronary artery bypass graft decreases graft patency compared with isolated coronary artery bypass graft: a meta-analysis. Interact Cardiov Th.

[5] Kelly JJ, Han JJ, Desai ND, Iyengar A, Acker AM, Grau-Sepulveda M (2022). Coronary endarterectomy: analysis of the Society of Thoracic Surgeons adult cardiac surgery database. Ann Thorac Surg.

[6] Walley VM, Byard RW, Keon WJ (1991). A study of the sequential morphologic changes after manual coronary endarterectomy. J Thorac Cardiovasc Surg.

[7] Kragel AH, McIntosh CM, Roberts WC (1989). Morphologic changes in coronary artery seen late after endarterectomy. Am J Cardiol.

[8] Roberts WC, Berry AE (2017). Frequency of coronary endarterectomy in patients undergoing coronary artery bypass grafting at a single tertiary Texas hospital 2010 to 2016 with morphologic studies of the operatively excised specimens. Am J Cardiol.

[9] Members WC, Lawton JS, Tamis-Holland JE, Bangalore S, Bates ER, Beckie TM (2021). ACC/AHA/SCAI guideline for coronary artery revascularization: a report of the American College of Cardiology/American Heart Association Joint Committee on Clinical Practice Guidelines. J Am Coll Cardiol.

[10] Schmitto JD, Kolat P, Ortmann P, Popov AF, Coskun KO, Friedrich M (2009). Early results of coronary artery bypass grafting with coronary endarterectomy for severe coronary artery disease. J Cardiothorac Surg.

[11] Marzban M, Karimi A, Ahmadi H, Davoodi S, Abbasi K, Movahedi N (2008). Early outcomes of double-vessel coronary endarterectomy in comparison with single-vessel coronary endarterectomy. Tex Heart Inst J.

[12] Kumar S, Agarwala S, Talbot C, Nair RU (2008). Long term survival after coronary endarterectomy in patients undergoing combined coronary and valvular surgery–a fifteen year experience. J Cardiothorac Surg.

[13] Kim JS, Han DC, Jeong YH, Park DW, Sohn CB, Hwang KW (2016). Antiplatelet effect of ticagrelor compared to tirofiban in non-ST-segment elevation ACS patients undergoing PCI. The result of the TE-CLOT trial. Thromb Haemostasis.

[14] Guo Y-Z, Zhao Z-W, Li S-M, Chen L-L (2021). Clinical efficacy and safety of tirofiban combined with conventional dual antiplatelet therapy in ACS patients undergoing PCI. Sci Rep-uk.

[15] Lincoff AM, LeNarz LA, Despotis GJ, Smith PK, Booth JE, Raymond RE (2000). Abciximab and bleeding during coronary surgery: results from the EPILOG and EPISTENT trials. Improve long-term outcome with abciximab GP IIb/IIIa blockade. Evaluation of platelet IIb/IIIa inhibition in STENTing. Ann Thorac Surg.

[16] Dyke CM, Bhatia D, Lorenz TJ, Marso SP, Tardiff BE, Hogeboom C (2000). Immediate coronary artery bypass surgery after platelet inhibition with eptifibatide: results from PURSUIT. Platelet glycoprotein IIb/IIIa in unstable angina: receptor suppression using integrelin therapy. Ann Thorac Surg.

[17] Bizzarri F, Scolletta S, Tucci E, Lucidi M, Davoli G, Toscano T (2001). Perioperative use of tirofiban hydrochloride (Aggrastat) does not increase surgical bleeding after emergency or urgent coronary artery bypass grafting. J Thorac Cardiovasc Surg.

[18] Djalilian AR, Shumway SJ (1995). Adjunctive coronary endarterectomy: improved safety in modern cardiac surgery. Ann Thorac Surg.

[19] Santini F, Casali G, Lusini M, D’Onofrio A, Barbieri E, Rigatelli G (2002). Mid-term results after extensive vein patch reconstruction and internal mammary grafting of the diffusely diseased left anterior descending coronary artery. Eur J Cardiothorac Surg.

[20] Takanashi S, Fukui T, Miyamoto Y (2008). Coronary endarterectomy in the left anterior descending artery. J Cardiol.

[21] Nishi H, Miyamoto S, Takanashi S, Minamimura H, Ishikawa T, Kato Y (2005). Optimal method of coronary endarterectomy for diffusely diseased coronary arteries. Ann Thorac Surg.

[22] Takahashi M, Gohil S, Tong B, Lento P, Filsoufi F, Reddy RC (2013). Early and mid-term results of off-pump endarterectomy of the left anterior descending artery. Interact Cardiovasc Thorac Surg.

[23] Tiemuerniyazi X, Yan H, Song Y, Nan Y, Xu F, Feng W (2021). Mid-term outcomes of coronary endarterectomy combined with coronary artery bypass grafting. Interact Cardiov Th.

[24] Dyke C, Aronson S, Dietrich W, Hofmann A, Karkouti K, Levi M (2014). Universal definition of perioperative bleeding in adult cardiac surgery. J Thorac Cardiovasc Surg.

[25] Mehran R, Rao SV, Bhatt DL, Gibson CM, Caixeta A, Eikelboom J (2011). Standardized bleeding definitions for cardiovascular clinical trials: a consensus report from the Bleeding Academic Research Consortium. Circulation.

[26] Thygesen K, Alpert JS, Jaffe AS, Chaitman BR, Bax JJ, Morrow DA (2018). Fourth Universal Definition of Myocardial Infarction (2018). J Am Coll Cardiol.

[27] Cooper WA, O’Brien SM, Thourani VH, Guyton RA, Bridges CR, Szczech LA (2006). Impact of renal dysfunction on outcomes of coronary artery bypass surgery: results from the Society of Thoracic Surgeons National Adult Cardiac Database. Circulation.

[28] Louagie YA, Haxhe JP, Jamart J, Buche M, Schoevaerdts JC (1994). Intraoperative assessment of coronary artery bypass grafts using a pulsed Doppler flowmeter. Ann Thorac Surg.

